# Next-generation sequencing identifies equine cartilage and subchondral bone miRNAs and suggests their involvement in osteochondrosis physiopathology

**DOI:** 10.1186/1471-2164-15-798

**Published:** 2014-09-17

**Authors:** Clémence Desjardin, Anne Vaiman, Xavier Mata, Rachel Legendre, Johan Laubier, Sean P Kennedy, Denis Laloe, Eric Barrey, Claire Jacques, Edmond P Cribiu, Laurent Schibler

**Affiliations:** INRA, UMR1313 Génétique animale et biologie intégrative, Domaine de Vilvert, 78350 Jouy-en-Josas, France; INRA, UMR1319 Micalis, Domaine de Vilvert, 78350 Jouy-en-Josas, France; UR4, University Pierre and Marie Curie, Paris, France; AgroParisTech, 16, rue Claude Bernard, 75231 Paris, France

**Keywords:** microRNA, Cartilage, Bone, Equine osteochondrosis

## Abstract

**Background:**

MicroRNAs (miRNAs) are an abundant class of small single-stranded non-coding RNA molecules ranging from 18 to 24 nucleotides. They negatively regulate gene expression at the post-transcriptional level and play key roles in many biological processes, including skeletal development and cartilage maturation. In addition, miRNAs involvement in osteoarticular diseases has been proved and some of them were identified as suitable biomarkers for pathological conditions. Equine osteochondrosis (OC) is one of the most prevalent juvenile osteoarticular disorders in horses and represents a major concern for animal welfare and economic reasons. Its etiology and pathology remain controversial and biological pathways as well as molecular mechanisms involved in the physiopathology are still unclear. This study aims to investigate the potential role of miRNAs in equine osteochondrosis (OC) physiopathology.

Short-read NGS technology (SOLID™, Life Technologies) was used to establish a comprehensive repertoire of miRNA expressed in either equine cartilage or subchondral bone. Undamaged cartilage and subchondral bone samples from healthy (healthy samples) and OC-affected (predisposed samples) 10-month Anglo-Arabian foals were analysed. Samples were also subjected or not to an experimental mechanical loading to evaluate the role of miRNAs in the regulation of mechano-transduction pathways. Predicted targets of annotated miRNAs were identified using miRmap.

**Results:**

Epiphyseal cartilage and subchondral bone miRNome were defined, including about 300 new miRNAs. Differentially expressed miRNAs were identified between bone and cartilage from healthy and OC foals, as well as after an experimental mechanical loading. In cartilage, functional annotation of their predicted targets suggests a role in the maintenance of cartilage integrity through the control of cell cycle and differentiation, energy production and metabolism as well as extracellular matrix structure and dynamics. In bone, miRNA predicited targets were associated with osteoblasts and osteoclasts differentiation, though the regulation of energy production, vesicle transport and some growth factor signaling pathways.

**Conclusion:**

Taken together, our results suggest a role of miRNAs in equine OC physiopathology and in the cellular response to biomechanical stress in cartilage and bone. *In silico* target prediction and functional enrichment analysis provides new insight into OC molecular physiopathology.

**Electronic supplementary material:**

The online version of this article (doi:10.1186/1471-2164-15-798) contains supplementary material, which is available to authorized users.

## Background

MicroRNAs (miRNAs) are an abundant class of small single-stranded non-coding RNA molecules of 18 to 24 nucleotides generated by a sequential processing of long RNA transcripts by two specific RNAse III proteins: Dicer and Drosha [1]. Studies performed during the past decade have led to the discovery of many miRNAs in almost all organisms [2]. Over 8000 predicted miRNAs from plants, animals and viruses are currently described in miRBase, the reference database. It has been shown that miRNAs negatively regulate gene expression at the post-transcriptional level by targeting specific mRNAs for cleavage or translational repression trough the RNA-induced silencing complex (RISC) [3]. They play key roles in many biological processes, including development, cell proliferation, differentiation as well as apoptosis and have been shown to be involved in a number physiopathological processes [4].

Analysis of cartilage-specific Dicer-knock-out (KO) mice [5] revealed a crucial role of miRNAs in cartilage development and integrity. However, a comprehensive identification of miRNAs expressed in cartilage and bone and their precise role is still unclear. Recent studies highlighted the role of some miRNAs in endochondral ossification [6], chondrocytes differentiation [7], regulation of bone remodeling [8] and osteoblast and osteoclast differentiation and functions [9–11]. In addition, miRNA expression profiling performed on bovine articular cartilage suggested their involvement in mechano-transduction pathway and in the maintenance of articular cartilage homeostasis [12]. In line with their role in cartilage maturation and bone development, miRNAs are now recognized as key players in osteoarthritis (OA) physiopathology [13, 14] and may prove to be involved in other osteoarticular diseases [15]. For example, it has been demonstrated that mir-27b regulates a protein involved in MEC turn-over, the Matrix Metalloproteinase 13 (MMP-13), in both normal and OA human chondrocytes. This result suggest that an abnormal expression of mir-27b may contribute to OA development [16]. Furthermore, some miRNAs have been identified as biomarkers for pathological conditions including OA [17].

Most recently, there has been growing interest in using next-generation sequencing (NGS) to catalog small RNAs in a variety of tissue. The aim of our study was to use short-read NGS technology (SOLID™, Life Technologies) to establish a comprehensive repertoire of miRNA expressed in either equine cartilage or subchondral bone and to investigate their potential role in equine osteochondrosis (OC). This juvenile osteoarticular disease leads to joint swelling, stiffness and varying degrees of lameness [18–20]. OC is one of the most prevalent developmental orthopedic diseases in equine population and constitutes a major concern in terms of animal welfare as well it economic impact [21]. Its etiology and pathology are still unclear, due to the confusion regarding the definition of the disease and the lack of understanding of primary lesions formation [22]. Clearly, there is a need for a better understanding of OC molecular physiopathology in order to refine nosological entities.

Here, we analysed undamaged cartilage and subchondral bone from healthy (healthy samples) and OC-affected (predisposed samples) foals, with and without an experimental mechanical loading. This was performed with the aim of exploring the role of miRNAs in the constitutive defects associated with OC development, as well as in regulating mechano-transduction pathways. This work made it possible to define, for the first time to our knowledge, the comprehensive epiphyseal cartilage and subchondral bone miRNome, including about 300 new putative miRNAs. Furthermore, we highlighted miRNAs associated with both OC status and mechanical loading, suggesting that miRNAs may be involved in OC physiopathology as well as in the cellular response to biomechanical stress. Some differentially expressed miRNA identified by our work may later prove to be valuable diagnostic biomarkers that could help refining the nosological entities.

## Methods

### Animal care committee

The experimental protocol was reviewed and approved under the number 0964 by the Animal Care Committee of VetAgro Sup, which abides by the requirements of the directive 86/609 of the European Community Council.

### Samples collection and experimental design

**Figure 1:**
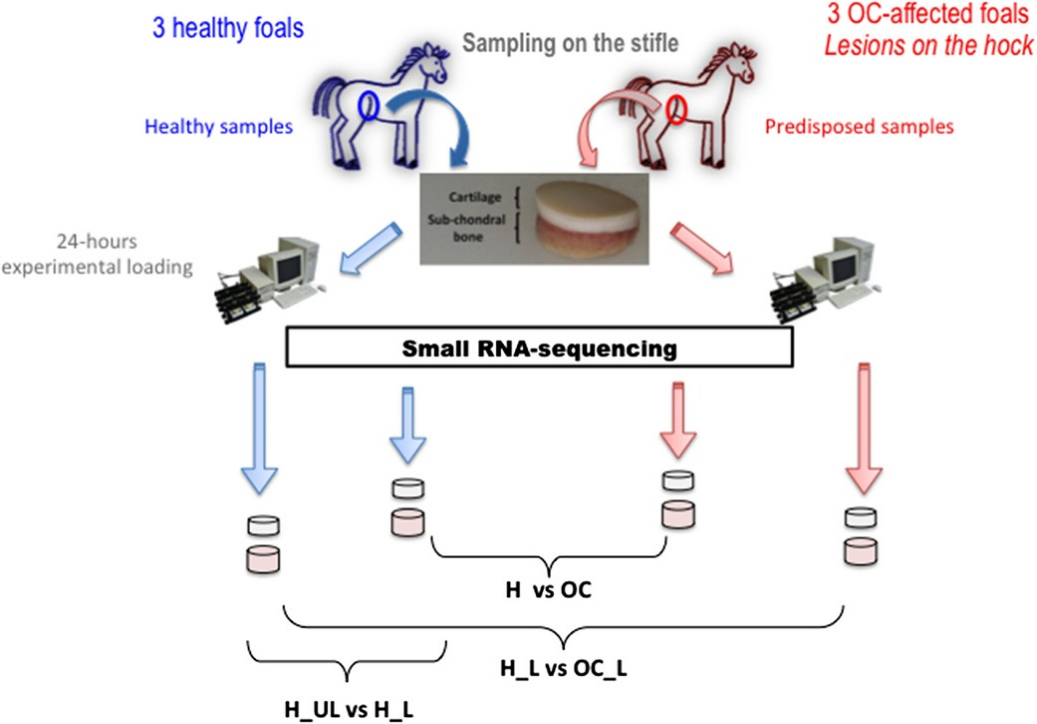
**Experimental design.** Three healthy foals and three OC-affected foals presenting lesion on the hock were divided in two groups. Healthy (H) and OC-predisposed (OC) cartilage and bone samples were pieced on the stifles. Samples were then subjected (_L) or not (_UL) to a 24-hours experimental loading. Short-read NGS technology (SOLID™, Life Technologies) was performed to define the cartilage and sub-chondral bone miRnomes. Three comparisons were used: between healthy and OC predisposed samples (H vs OC) to test the hypothesis of a constitutive defect, between healthy loaded and unloaded samples (H_L vs H_UL) to evaluate the role of miRNA in the response to biomechanical stress and between healthy and OC-predisposed samples loaded (H_L vs OC_L) to test the hypothesis of a impaired response to mechanical loaded in OC-affected foals.

The study was based on 37 Anglo-Arabian foals obtained from a unique stallion known to have OC-affected foals in his progeny (Figure 1). The osteo-articular status of the mares was not extensively ascertained, but none was diagnosed as OC-affected based on clinical examination.

Foals were bred at the experimental station of the French National Stud (Station IFCE of Chamberet) to reduce genetic and environmental variability. Mare-foal couples were managed on swivel pastures until the end of the grass season. The osteo-articular status of each foal was determined between six to seven months of age to reliably identify OC lesions. Horses were subjected to a clinical examination consisting in a qualitative visual assessment for lameness, trauma, skin anomalies and joint effusion. An X-ray examination of fetlock, tarsocrural and stifle joints of each foal was then performed and images were evaluated for the presence of OC signs. If irregularities or anomalies were noted, additional X-rays were taken to confirm the lesion. Foals were considered healthy if the clinical exam was normal without ambiguity and if X-rays did not reveal any sign of osteo-articular irregularities. Three healthy and three OC-affected (at least one lesion on the hock) foals were selected and euthanized at 10 months by lethal intravenous injection of T61 (Embutramide), a sedative drug used for euthanasia in veterinary medicine. All joints were macroscopically examined at necropsy to ensure the healthy status and confirm radiologic diagnosis. Cartilage and bone cylindrical explants were obtained from the stifle (healthy joint) using a 12-mm punch. Samples were collected on the same location in the middle of the trochlea to avoid technical bias due to sample location in the joint. Explants including epiphyseal cartilage and about 5 mm of the soft underlying bone (considered as subchondral bone) were washed in PBS and placed in DMEM culture medium for subsequent mechanical-loading experiments or flash frozen in liquid nitrogen for subsequent RNA-seq analysis.

OC-lesions were also collected, fixed for 24 hours in a solution of 4% paraformaldehyde, decalcified in EDTA 10% pH 8.8 solution for one month before being embedded in paraffin and sectioned (5 μm). Sections were stained with safranin-O and light green (Additional file 1).

### Experimental loading

Harvested cartilage explants were placed into individual compression wells of Biopress culture plates (Flexercell International) in 1.5 ml of culture medium (DMEM, containing penicillin-streptomycin 1%, glutamine 2%, albumin 0.1% and Hepes 30 mM). All experiments were performed at 37°C, under ambient atmosphere. A compressive stress was applied to individual samples by the Flexercell FX-4000C system (sinusoidal waveform at 0.5 Hz, 1MPa, 24h00) whereas the control explants stayed in unloaded conditions, as previously described [23].

Protaglandin E2 (PGE2) production was measured in the media by a high sensitivity commercially available enzyme immunoassay kit (Cayman Chemical), as previously described [24].

### Sequencing

miRNA was extracted from ~150mg of tissue (bone and cartilage) which had been previously frozen at -80°C post-necropsy and subsequently stored in liquid nitrogen. The *mir*Vana™ miRNA Isolation Kit (Ambion, Life Technologies) was used to extract in parallel Total RNA and small RNA (smRNA) from cartilage following the manufacturer’s protocol. Total RNA was purified from bone using the miRNeasy Mini kit (Qiagen) and the RNeasy MinElute Cleanup kit (Qiagen) for enrichment of small RNA (<200 nt) following the manufacturer’s protocol. Total RNA quality was assessed using an Agilent Bioanalyzer 2100 (Agilent Technologies) loaded with a RNA 6000 Nano Kit. The concentrations of smRNA were determined using a NanoDrop ND-1000 Spectrophotometer and the size and purity were determined using the Bioanalyzer 2100 loaded with a RNA 6000 Nano Kit. Approximately 190ng of miRNA extracted from bone and 70ng from cartilage was used for library constructions following the protocol for the SOLiD® Total RNA-Seq Kit (Applied Biosystems, Life Technologies). A total of 30 libraries were prepared: 15 for cartilage and 15 for bone. Among these 15 libraries, 3 were prepared from healthy cartilage/bone from healthy horses, 3 from healthy cartilage/bone from OCD horses, 3 from healthy cartilage/bone from healthy horses after 24H of compression, 3 from healthy cartilage/bone from OCD horses after 24H of compression and 3 from healthy cartilage/bone from healthy horses in the culture medium used during compression.

Briefly, the small RNAs were hybridized and ligated with SOLiD™ Adaptor Mix, a reverse transcription was performed and the cDNAs were purified using the MinElute PCR purification kit (Qiagen). The 60–80 base pair fraction of the cDNA was excised from a 10% acrylamide gel stained with SYBR Gold (Invitrogen, Life Technologies) and an amplification was realized by PCR (95°C - 1 min, 18 × (95°C -30s, 62°C – 30 s, 72°C – 30 s), 72°C – 7 min) using the SOLiD 5’ PCR primer and a 3’ library-specific barcoded SOLiD primer. The amplified DNA was purified using the Purelink PCR micro kit (Invitrogen, Life Technologies) and the 110–130 bp region was excised from a 6% acrylamide gel stained with SYBR Gold (Invitrogen, Life Technologies). The yield and size distribution of the amplified DNA were assessed and pools of three different horses were made to decrease the effect of individual variability. Libraries were processed on the SOLiD EZBead system for template bead preparation prior to sequencing.

Small RNA libraries were sequencing on a SOLiD 5500XL Series Genetic Analysis System at the Metaquant Platform (INRA, Jouy-en-Josas). 350 million 50-nt reads were generated over the thirty libraries.

### Sequence data deposition

Raw sequence data and protocol details were submitted to the ArrayExpress database (http://www.ebi.ac.uk/arrayexpress/) through the Annotare 2.0 submission tool. The experiment has been assigned accession number E-MTAB-2736.

### Adaptor trimming and reads mapping

Adaptors in color space (5’-330201030313112312-3’) were removed by Cutadapt (version 0.9.5), permitting two mismatches over reads between 17 and 26 nucleotides. Read mapping was performed using Bowtie (version 0.12.7) with color space options, in v- alignment mode, permitting 2 mismatches. Uniquely mapping reads and reads with <6 total alignments were retained. In the second case, all alignments were reported in the best-to-worst order (*best* option). Reads with >6 alignments were removed. Mapped reads overlapping with known non-coding RNAs (except miRNAs) obtained from RFAM (http://rfam.sanger.ac.uk/) were also discarded.

### *Loci*detection and quantification

Samtools (http://samtools.sourceforge.net/) was used to convert the SAM files to BAM files and Bedtools (http://code.google.com/p/bedtools/) to retrieve information from the alignment file and extract it into a *.wig file with all positions available (chromosome, start, stop) for each read or *locus* mapped to the reference genome (“genomeCoverageBed”). The candidate *loci* were then used as guidelines for excising potential miRNAs precursor sequences from the horse genome. Two potential sequences were excised, one at -10 bp and +50bp centered on the island and another at -50bp and +10bp, to simulate the 5p and 3p position of the potential miRNA. Each potential precursor sequence was passed to RNAfold tool (Vienna package, version 1.8.5) to predict secondary structures and detect potential hairpin structure. In-house Perl scripts were used to count reads in each candidate *loci* for all samples.

### miRNAs annotation

Equine mature miRNAs annotations were extracted from the miRBase database (http://www.mirbase.org/). In order to extend annotations, human, bovine, dog, pig and mouse miRNAs precursor sequences were aligned to the horse genome by BLAST (version2.2.25). Hits with a bit score higher than 90 were conserved and IntersectBed (tool of BedTools) was used to annotate each candidate miRNA. Manual cleaning and curation was performed to correct or improve nomenclature.

For putative new miRNAs, we applied the follow filtering criteria: a size between 17 and 29 bases, a count greater than or equal to 1000 in at least one experimental library and a free energy of the secondary structure calculated by RNAfold lower than -20 kcal/mol, which is characteristic of known miRNAs RNAfold scores. MapMi (http://www.ebi.ac.uk/enright-srv/MapMi/) was used to query orthologs in five other mammalian species (cattle, pig, humans, dog and mouse).

### Statistical analysis

Statistical analysis for determining differential expression was performed with R v2.15.0 [25] using the Bioconductor package DESeq version 1.0.0 [26]. DESeq utilizes a negative binomial distribution for modeling read counts per miRNA and implements a method for normalizing the counts. This normalization procedure uses the library median of the ratios between the read count and the geometric mean of each gene as a scaling factor for each library. The p-values were adjusted for multiple testing using the Benjamini and Hochberg method [27]). A comprehensive description of miRNA expression patterns was performed by principal component analysis (PCA) and between-class analysis (PCA of the table of the group means, BCA). The R software (version 1.4.17) was used with the R package ade4 *(*Version 1.5-2*)*.

### Validating RNA-Seq data using Taqman qPCR technology

Total RNA was extracted from cartilage (mirVana™ miRNA Isolation Kit Ambion, Life Technologies) and bone (miRNeasy Mini Kit, Quiagen) samples according to the instructions of the respective extraction kits. For each samples, 10 ng of total RNA were obtained. The RNA was reverse transcribed into cDNA using the additional poly(A) tail reaction method according to the instructions of the reverse transcription kit (TaqMan® MicroRNA Reverse Transcription Kit, Applied Biosystems, Life Technologies). The qPCR reaction was performed according to the instructions of the qPCR kit (TaqMan® Universal Master Mix II, Applied Biosystems, Life Technologies) and a Mastercycler Ep Realplex apparatus (Eppendorf). For each cartilage and bone samples, reactions were performed in triplicate. Results were analysed using the qBase + data analysis software and expressed as normalized relative quantity (NRQ) [28] based on one reference gene for each tissue. Reference genes were chosen based on RNA-seq results, as having the smallest variations between all samples from each tissue.

### Identification and functional annotation of miRNAs predicted targets

The miRmap web tool (http://mirmap.ezlab.org/app/) was used to query for miRNAs predicted targets [29]. For each miRNAs of interest, a comprehensive target prediction was obtained. We considered only predicted targets having a miRmap score above 90 and retained, at most, the first 500 with the best score. In addition, these targets were also filtered based on known expression patterns in cartilage and bone, respectively. Functional annotation was performed using PANTHER (http://www.pantherdb.org/) [30] and comparative statistical enrichment analysis was done using ToppCluster (http://toppcluster.cchmc.org/).

## Results

### Experimental mechanical loading

Two explants from each horse were subjected to compressive stress using a Flexercell FX-4000C system, whereas two other explants were left in unloaded conditions to serve as controls. Prostaglandin E2 (PGE2) release in the media (pg/mg/ml of explant) was measured to evaluate compression efficiency. In agreement with previous data obtained on mouse costal cartilage explants [31], a significant two-fold induction was observed, on average, between compressed and uncompressed samples (Supplementary data). No significant differences were observed between compressed predisposed and healthy samples.

### Identification and characterization of miRNAs in equine bone and cartilage tissues

Total RNA and small RNA were extracted in parallel. RIN between 7.5 and 8 were observed for cartilage samples, whereas RIN in the range 8.5 to 9 were obtained for bone samples. Thirty libraries were built and sequenced using the SOLID™ technology, generating 9 to 15 million reads for each library. All sequence data can be retrieved from the ArrayExpress database under accession number E-MTAB-2736. After adaptor trimming and size selection, about 70% of these reads were mapped to the EquCab2 horse genome for each sample, defining about 53998 to 97964 regions. Among these regions, 327 and 329 in cartilage and bone, respectively, overlapped with previously known mammalian miRNA (Additional file 2: Table S1-A, and Additional file 3: Table S1-B). Secondary structure analysis identified 282 and 293 putative new miRNAs in cartilage and bone, respectively (Supplementary data). Only regions with a genomic coverage greater than 2 were kept for further analysis.

We elaborated a set of 609 expressed miRNAs in cartilage and 622 miRNAs in bone. 561 miRNAs were found to be expressed in both tissues.

About 300 putative new miRNAs were identified based on their expression level and RNAfold score. Existing miRNA precursor sequences were searched using MapMi in five other mammalian genomes (cattle, pig, humans, dog and mouse), identifying likely orthologs in at least one species for 163 miRNAs.

### Differential miRNAs expression analysis

**Figure 2:**
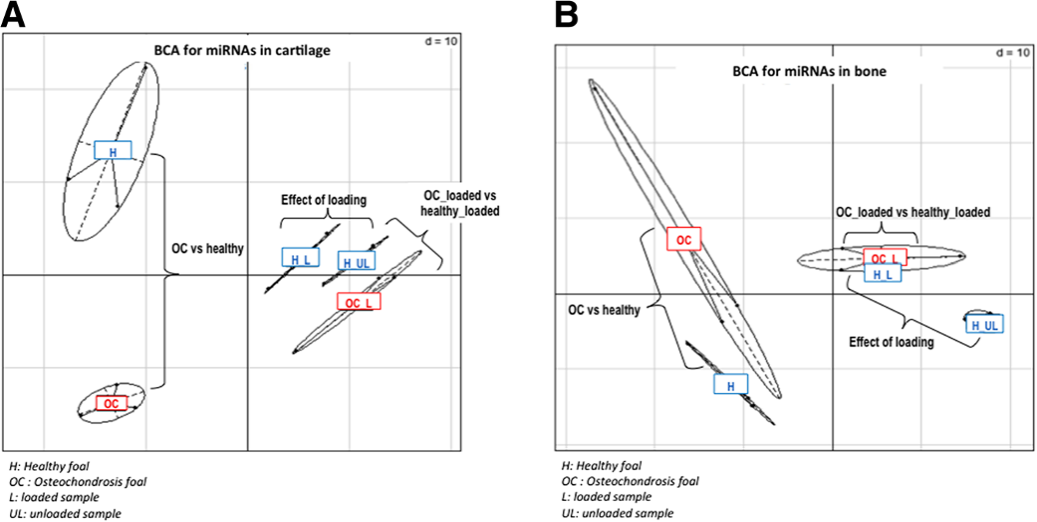
**Between Class Analysis (BCA) based on A- cartilage and B- bone miRnomes.** BCA succeed in discriminating between healthy samples (H) and predisposed samples of OC-affected foals (OC), as well as healthy samples loaded (H_L) and unloaded (H_UL) in both cartilage and bone. This suggests that miRNA may be involved in OC physiopathology and biological response to mechanical loading. In contrast, BCA does not well discriminate between healthy samples loaded (H_L) and OC predisposed samples loaded (OC_L) in cartilage and bone. This may prove that biomechanical response is not disrupted in cartilage and bone predisposed samples of OC-affected foals.

A between-class correspondence analysis was performed for each tissue, showing a good discrimination between each experimental condition (Figure 1): normal native cartilage or bone from healthy (H) *versus* affected (OC) foals, before (H_UL) *versus* after (H_L, OC_L) an experimental 24h mechanical loading (Figure 2 A-B). Differential analyses were also performed for each tissue and each condition. Loaded and unloaded samples could be efficiently discriminated, confirming the efficiency of the experimental compression.

**Figure 3:**
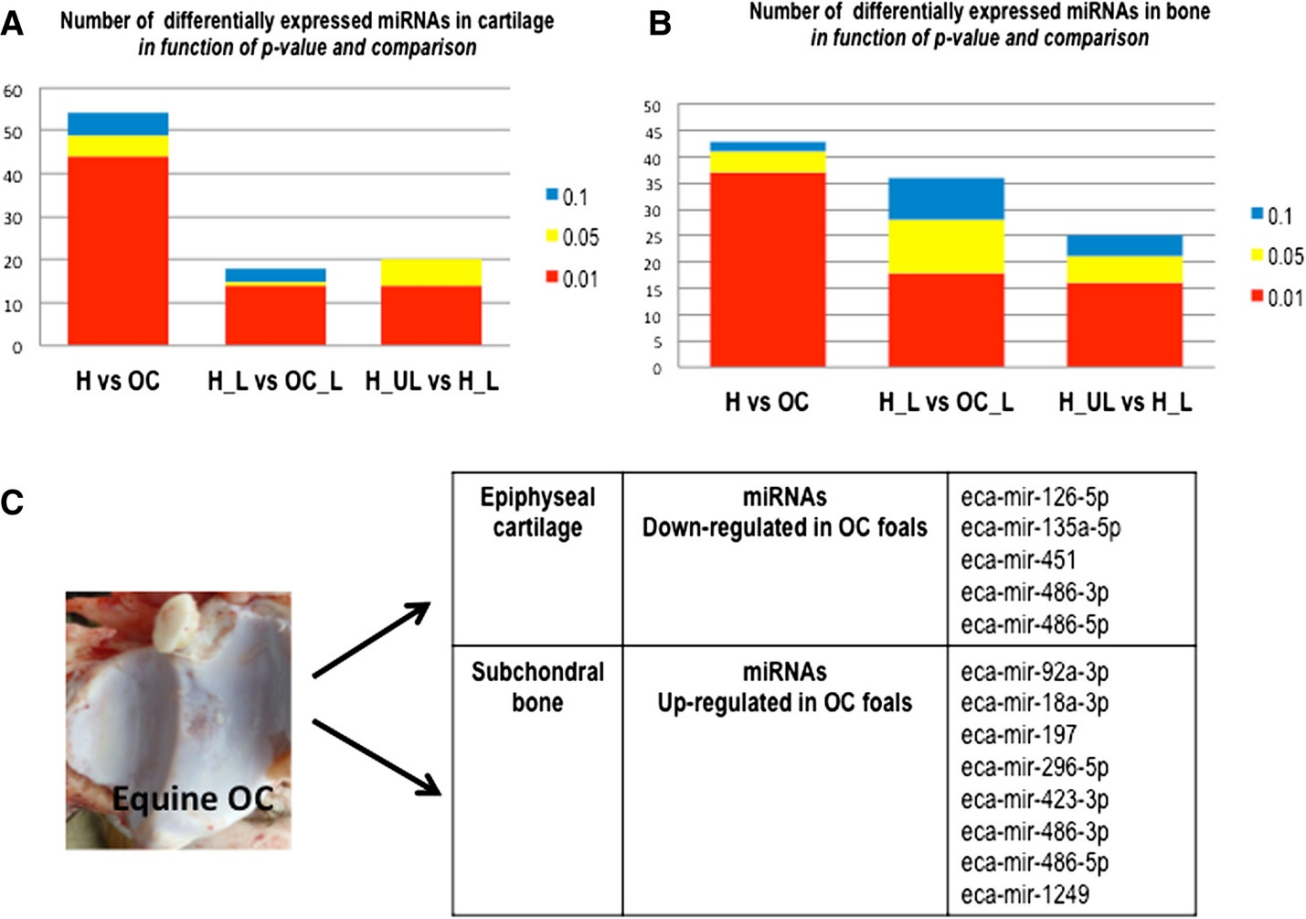
**Number of differentially expressed miRNAs in cartilage A- and bone B- according to** ***p-value*** **(0,1; 0,05; 0,01) and comparisons performed: between healthy and OC predisposed samples (H vs OC); between healthy loaded and unloaded samples (H_L vs H_UL); between healthy and OC-predisposed samples loaded (H_L vs OC_L).** C- Annotated miRNAs differentially expressed between healthy and predisposed cartilage and bone. In cartilage, 5 annotated miRNAs were down-regulated whereas in bone, 8 annotated miRNAs were up-regulated. Those data suggest a role of miRNAs in OC physiopathology.

Expression levels of miRNA in normal bone and cartilage from healthy and predisposed samples were compared to determine the potential role of miRNAs in equine OC physiopathology. This analysis highlighted 49 miRNAS differentially expressed in cartilage (including 5 annotated) and 41 miRNAs (8 annotated) in bone (Figure 3-A,B,C). In cartilage, 3 miRNA were found to be up-regulated in contrast to bone where 40 miRNAs were up-regulated and only a single miRNA species was found to be down-regulated (Figure 3-A, B,C). Six differentially expressed miRNAs (three in each tissue) were also studied by qPCR on a panel of 5 OC-affected and 5 healthy foals to confirm RNA-seq results. Expression and differential trends were confirmed in all cases, but 2 miRNAs failed to reach statistical significance (supplementary data).

We also investigated changes in miRNA expression induced by mechanical stress. Nineteen differentially expressed miRNAs were identified in cartilage (2 annotated) and 21 in bone (2 annotated). Among these, 12 miRNAs were up-regulated in cartilage and 9 in bone (Figure 3-A-B).

Finally, in order to test the hypothesis of an impaired response to biomechanical stress in OC horses, miRNA expression was compared in normal bone and cartilage from OC *versus* healthy foals after experimental loading. In cartilage, 15 miRNAs (1 annotated) were modulated, 7 of these being up-regulated (Figure 3-A). In bone, 28 miRNAs (2 annotated) were differentially expressed, including 24 up-regulated and 1 down-regulated in OC foals (Figure 3-B).

### Identification and functional annotation of miRNAs predicted targets

**Figure 4:**
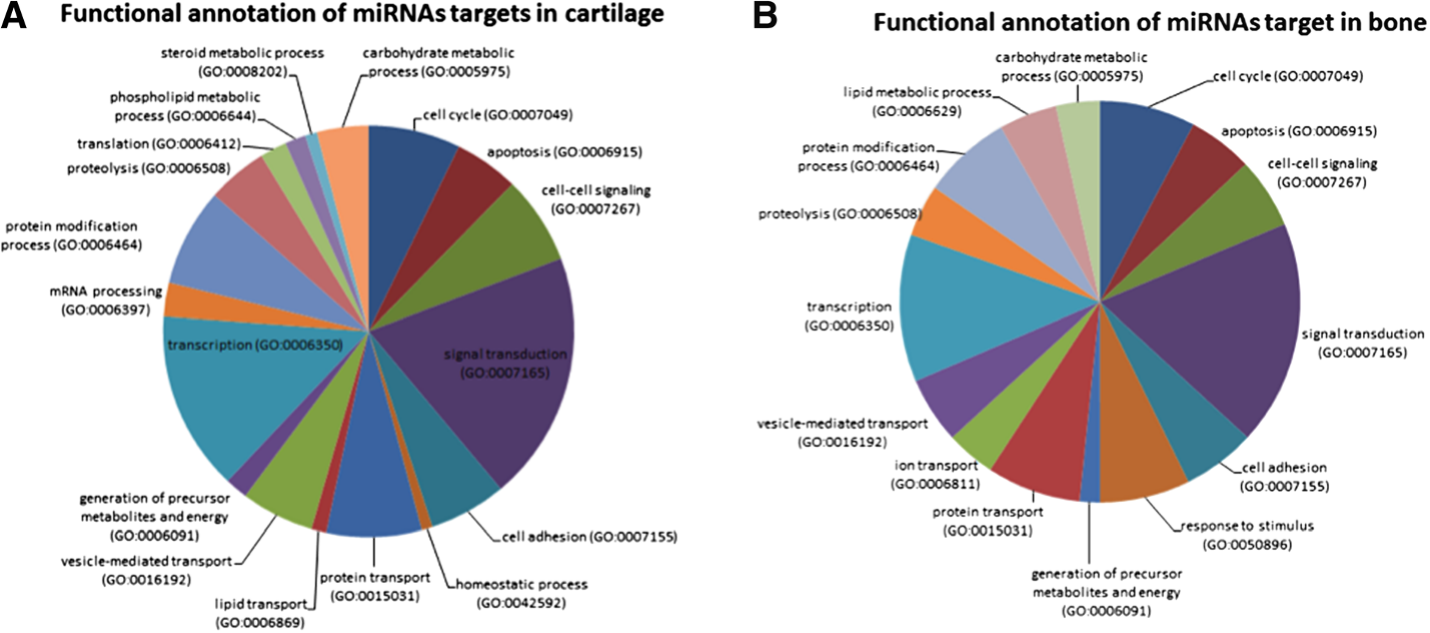
**Molecular functions of targets of miRNAs differentially expressed between healthy and OC-affected foals in cartilage (A, upper panel) and bone (B, lower panel).** The diagrams show the proportion of modulated miRNAs targets belonging to the most significant functional annotations derived from the Gene Ontology (GO).

Predicted targets of annotated miRNAs differentially expressed in bone and cartilage of OC foals were queried using miRmap [29]. About 2400 putative targets could be identified, ranging from 42 to 250 targets for each miRNAs of interest in cartilage and from 51 to 250 in bone (177 and 192 targets on average in cartilage and bone, respectively). Global functional annotation using PANTHER showed that predicted targets were primarily involved in cell cycle, cell adhesion, energy production and metabolism, as well as cell communication, adhesion and transport (Figure 4A and B). Functional enrichment analysis in cartilage highlighted genes primarily associated with skeletal phenotypes and involved in cell cycle and differentiation, energy production and metabolism (ATPase and GTPase activity, carbohydrate metabolic process, macromolecule biosynthesis), proteins modifications, folding and transport (protein modification process, transport, endoplasmic reticulum unfolded protein response, proteolysis), cellular homeostasis, extracellular matrix structure and dynamics (collagen, chondroitin sulfate and glycosaminoglycan catabolic process, regulation of metalloendopeptidase activity), cellular organization (cytoskeleton), ossification as well as regulation of Wnt and fibroblast growth factor signaling pathways (Additional file 2).

In bone, genes were also associated with skeleton development and morphogenesis, as well as osteoblasts and osteoclasts differentiation (Additional file 3). Enriched functions included energy production (Ras and Rab GTPase, mitochondria), calcium homeostasis (endoplasmic reticulum calcium ion homeostasis, calcium channel activity), lipid biosynthesis and transport, vesicle transport (vesicle organization, vesicle-mediated transport, Golgi to ER retrograde vesicle-mediated transport, endocytosis, exocytosis), as well as regulation of growth factor signaling pathways (fibroblast growth factor, insulin-like growth factor I, transforming growth factor beta).

## Discussion

In this study we characterized miRNA expression profiles in equine epiphyseal cartilage and subchondral bone using NGS analysis. We identified 609 and 622 miRNAs expressed in cartilage and bone, respectively, including about 300 novel miRNAs. Recently, Sun *et al*., described the use of a Solexa-based deep sequencing approach to identify miRNAs profiles of articular cartilage from rat femoral head cartilage. This study made it possible to highlight 310 known miRNAs as well as 86 novel miRNAs candidates [32]. Since 215 miRNA are found in common with the present study, our results extend the repertoire of miRNA expressed in cartilage by adding 111 additional miRNAs. We provide a comprehensive repertoire of miRNAs expressed in both cartilage and bone samples collected *in vivo*. In particular, our results highlighted numerous new putative highly expressed miRNAs based on a high confidence RNA-fold score. Most of these also display orthologous precursor sequences in at least one other mammalian genome. Furthermore, they were found to be modulated between healthy and OC-affected horses, providing support for the hypothesis that these sequences do not account for experimental artifacts, but rather may represent genuine miRNAs or smallRNAs. Such a large number of new putative miRNAs may be due to the lack of studies and data specifically targeting cartilage and bone miRnome.

Equine osteochondrosis is the most common developmental osteoarticular disorder in equine populations leading to pain and lameness. Primary lesions are thought to results from a focal disruption of endochondral ossification, leading to cartilage retention into subchondral bone. Several studies have suggested that primary lesions result from an initial dyschondroplasia associated with an impaired differentiation of chondrocytes [22, 33] whereas other studies have supported the hypothesis of an initial disruption of canal blood supply leading to the formation of necrotic cartilage areas [34–36]. Here, our miRNome analysis agrees with the hypothesis of a constitutive defect leading to impaired cartilage maturation. Indeed, our study was focused on undamaged samples collected on stifle from healthy foals and foals affected by OC lesions on hock. Differential analysis highlighted 49 and 41 modulated miRNAs in cartilage and bone, respectively. Interestingly, several gene mapping programs have been initiated worldwide in different breeds and several quantitative trait loci (QTL) regions have been identified (for review see [37]). On these regions, two were found to contain a new miRNA differentially expressed in cartilage (new-eca-mir-696 and new-eca-mir-754) and one overlap with the location of eca-mir-1249, which has been shown to be modulated in bone (Additional file 4).

In cartilage, predicted target genes were found associated with known functions and mechanisms involved in maintenance of cartilage integrity. For example, the balance between catabolic and anabolic processes plays a critical role in cartilage homeostasis [38]. It has been shown that several miRNAs including mir-140, mir-199a, mir-193 and mir-29a/29b may be involved in cartilage anabolic and catabolic regulation by regulating the expression of key genes involved in those process such as MMP13, Adamts-5 or Col2A1 (for review see [39]). Following these results, our data suggest that differentially expressed miRNAs regulate genes involved in macromolecule metabolism and protein folding and transport. This may also reflect a modification of cartilage turn-over in OC foals. Furthermore, chondrocyte energy metabolism plays a major role in chondrocyte development, maturation and mineralization process [40–42]. Likewise, actin cytoskeleton, which plays a major role in chondrocyte hypertrophic differentiation [43], was highlighted in our data. Taken together, our results suggest that down-regulation of miRNAs expression in OC predisposed foals may alter pathways crucial for cartilage maturation, leading to an abnormal extracellular cartilage matrix synthesis. Interestingly, extracellular matrix changes were observed in OC-affected horses, often associated with a disruption of cartilage canals [33, 44–46]. Thus, our findings are consistent with a role of miRNAs in cartilage genetic susceptibility to equine OC.

Likewise, in bone, miRNAS target genes were found associated with functions essential for bone development and remodeling, as well as bone cell differentiation and activation. For instance, energy production and metabolisms are undoubtedly essential for proper bone tissue development. Indeed, ATP is essential for osteoclasts to generate the acidic environment required for solubilization of the alkaline salts and digestion of the organic bone matrix [47], and is involved in osteoblast differentiation and activation [48]. In addition, several studies have demonstrated that Rab GTPases, a family of proteins involved in membrane traffic, play an key role for osteoclasts function, in particular for the polarized transport of acidic vesicles of the endocytic/lysosomal pathway required for formation of the ruffled border (for review see [49]). Likewise, lipids, especially cholesterol, are essential for osteoblasts and osteoclasts development (for review see [50]). Consistent with the known role of miRNAs in the control of bone remodeling (for review see [8]) and their involvement in key regulation pathways of bone formation and remodeling such as BMP-signaling pathway (mir-133, mir-135) [51] and Wnt-signaling pathway (mir-29a) [52], miRNA predicted targets were found to be involved in the regulation of several signaling pathways (FGF, TGFb, IGF1). An essential role of zinc finger proteins in the regulation of osteoblast differentiation and bone remodeling has also recently emerged. For example, ZFP521 acts as a co-factor of Runx2 and the Runx2/ZFP521 balance regulates bone homeostasis [53]. ZNF384 regulates expression of type I collagen, counteracts BMP2-enhanced osteoblast differentiation as well as PTH induced MMP-13 expression [54]. Our data supports a role of miRNA in a constitutive bone defect associated with a predisposition to OC, including impaired bone remodeling and bone cells homeostasis.

To investigate the role of miRNAs in mechanotransduction pathways in bone and cartilage, samples were subjected to a 24-hours experimental loading treatment. The comparative analysis between unloaded and loaded samples made it possible to identify modulated miRNAs in cartilage and in bone. Regulation of mechanotransduction pathways in articular cartilage by miRNAs was previously suggested in a study performed with bovine articular cartilage [12]. This study allowed for the identification of additional miRNAs (mir-17-3p, mir-874-3p) as well as 17 new ones involved in response to mechanical stress. This work is the first to demonstrate a role of miRNAs in the regulation of mechanotransduction pathways in bone.

## Conclusions

In conclusion, based on the miRNome, equine OC appears to be associated with combined cartilage and bone defects, in good agreement with our previous preliminary proteomics findings [55]. The precise mechanisms linking cartilage and bone defects remain to be elucidated. Moreover, cartilage and subchondral bone serve as a functional unit to maintain joint homeostasis and a disruption or modification of either of these two tissues may lead to the remodeling of the other (for review see [56]). Nevertheless, factors involved in this interaction remain unknown. miRNAs are secreted in both tissues and may able to take part in the communication from cartilage to bone and from bone to cartilage through the synovial fluid. In fact, miRNAs are present in synovial fluid and their expression is modified in pathological situations such as OA [17]. We observed miRNAs differentially expressed between healthy and OC cartilage and bone, some of which should constitute relevant OC biomarkers in synovial fluid and, to a lesser extent, in plasma.

Finally, there is no evidence indicating that identified defects could alone be sufficient to induce OC lesions. Since biomechanical constraints are thought to trigger the development of focal lesions, we also explored the hypothesis of an impaired response to biomechanical constraint and some mild differences could be observed between healthy and predisposed samples. However, it remains unclear whether these differences may reflect a constitutively impaired response to biomechanical constrains or may reflect changes in ECM structure and biomechanical properties, consequently modifying mechanical signal transduction and leading to a slightly different outcome.

## Electronic supplementary material

Additional file 1: Figure S1: Histological analysis of OC lesions. Upper Panels - Macroscopic views of the *talus trochlea* of one healthy and the three OC-affected foals. The presence of OC-lesions is indicated by an arrow. Lower Panels - Histological views of OC lesions (light green/safranin-O stain). The light green stains the type-II collagen making it possible to reveal bone tissue whereas safranin-O marks proteoglycans revealing the cartilage. Osteochondral lesions for histological analysis were cut in the sagittal plane to include 5 mm of subchondral bone and fixed for 18 hours in a solution of 4% paraformaldehyde (PFA), decalcified for one month in 20 ml DC3 solution and embedded in paraffin. 5-μm sections were stained with safranin O-Light Green (LGS). Irregularities and reduced thickness were observed at the cartilage surface. The absence of staining both in and close to the lesion indicates reduction in proteoglycan content suggesting that cartilage is composition is modified. Abnormal cartilage cores were also observed into the subchondral bone which may reflect an abnormal bone maturation process. (TIFF 17 MB)

Additional file 2: **Cartilage mirnome and differential expression analysis between cartilage from healthy and OC horse.** (XLSX 228 KB)

Additional file 3: **Bone mirnome and differential expression analysis between cartilage from healthy and OC horse.** (XLSX 284 KB)

Additional file 4: **QTLs associated with equine osteochondrosis and miRNAs localization.** (XLSX 10 KB)
