# Supplementary figures and images for: Next-generation sequencing identifies equine cartilage and subchondral bone miRNAs and suggests their involvement in osteochondrosis physiopathology
Source: BMC Genomics. 2014 Sep 17;15(1):798. doi: 10.1186/1471-2164-15-798 (PMC4190437; doi:10.1186/1471-2164-15-798)

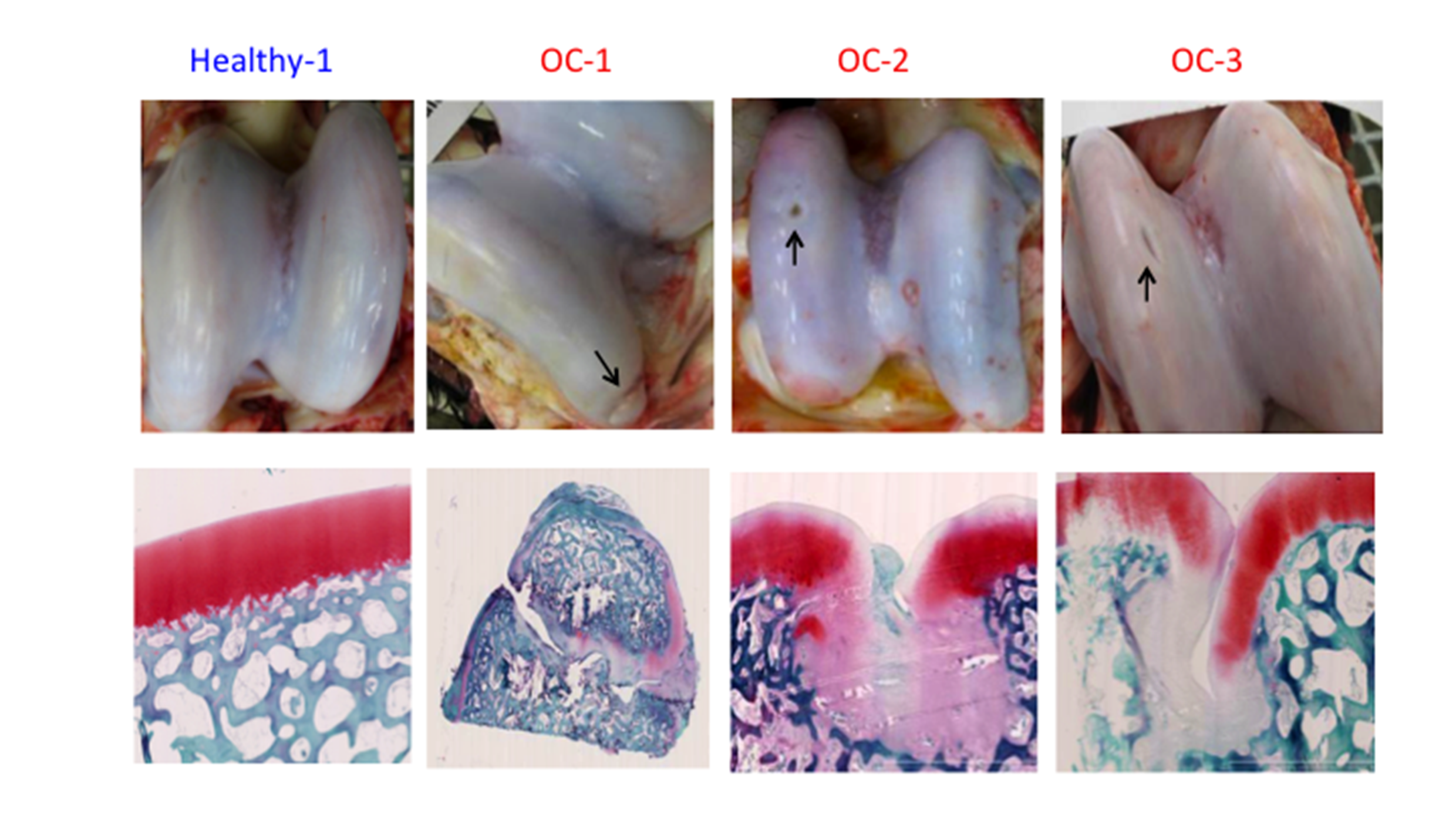

Supplement: Supplementary file 1 — Additional file 1: Figure S1: Histological analysis of OC lesions. Upper Panels - Macroscopic views of the talus trochlea of one healthy and the three OC-affected foals. The presence of OC-lesions is indicated by an arrow. Lower Panels - Histological views of OC lesions (light green/safranin-O stain). The light green stains the type-II collagen making it possible to reveal bone tissue whereas safranin-O marks proteoglycans revealing the cartilage. Osteochondral lesions for histological analysis were cut in the sagittal plane to include 5 mm of subchondral bone and fixed for 18 hours in a solution of 4% paraformaldehyde (PFA), decalcified for one month in 20 ml DC3 solution and embedded in paraffin. 5-μm sections were stained with safranin O-Light Green (LGS). Irregularities and reduced thickness were observed at the cartilage surface. The absence of staining both in and close to the lesion indicates reduction in proteoglycan content suggesting that cartilage is composition is modified. Abnormal cartilage cores were also observed into the subchondral bone which may reflect an abnormal bone maturation process. (TIFF 17 MB) [file 12864_2013_6497_MOESM1_ESM.tiff]
